# Comparative Analysis of Early Life Stage Traits in Annual and Perennial *Phaseolus* Crops and Their Wild Relatives

**DOI:** 10.3389/fpls.2020.00034

**Published:** 2020-03-10

**Authors:** Sterling A. Herron, Matthew J. Rubin, Claudia Ciotir, Timothy E. Crews, David L. Van Tassel, Allison J. Miller

**Affiliations:** ^1^ Department of Biology, Saint Louis University, St. Louis, MO, United States; ^2^ Donald Danforth Plant Science Center, St. Louis, MO, United States; ^3^ The Land Institute, Salina, KS, United States

**Keywords:** perennial grain, *Phaseolus*, Fabaceae, legume, pulse, crop wild relative, domestication

## Abstract

Herbaceous perennial species are receiving increased attention for their potential to provide both edible products and ecosystem services in agricultural systems. Many legumes (Fabaceae Lindl.) are of special interest due to nitrogen fixation carried out by bacteria in their roots and their production of protein-rich, edible seeds. However, herbaceous perennial legumes have yet to enter widespread use as pulse crops, and the response of wild, herbaceous perennial species to artificial selection for increased seed yield remains under investigation. Here we compare cultivated and wild accessions of congeneric annual and herbaceous perennial legume species to investigate associations of lifespan and cultivation with early life stage traits including seed size, germination, and first year vegetative growth patterns, and to assess variation and covariation in these traits. We use “cultivated” to describe accessions with a history of human planting and use, which encompasses a continuum of domestication. Analyses focused on three annual and four perennial species of the economically important genus *Phaseolus*. We found a significant association of both lifespan and cultivation status with seed size (weight, two-dimensional lateral area, length), node number, and most biomass traits (with cultivation alone showing additional significant associations). Wild annual and perennial accessions primarily showed only slight differences in trait values. Relative to wild forms, both cultivated annual and cultivated perennial accessions exhibited greater seed size and larger overall vegetative size, with cultivated perennials showing greater mean trait differences relative to wild accessions than cultivated annuals. Germination proportion was significantly lower in cultivated relative to wild annual accessions, while no significant difference was observed between cultivated and wild perennial germination. Regardless of lifespan and cultivation status, seed size traits were positively correlated with most vegetative traits, and all biomass traits examined here were positively correlated. This study highlights some fundamental similarities and differences between annual and herbaceous perennial legumes and provides insights into how perennial legumes might respond to artificial selection compared to annual species.

## Introduction

Life history theory traditionally categorizes plants as annuals, typified by fast growth and high reproductive effort, and perennials, typified by long-term survival and delayed reproduction (Cole, 1954; Harper, 1977). However, exceptions to this general trend occur; patterns of plant resource allocation exist along a gradient, with some perennial species showing fast growth and high reproductive output (e.g., Verboom et al., 2004; González-Paleo and Ravetta, 2015). Exploration of this life history diversity is important in plant breeding, as wild, herbaceous perennial species are increasingly considered as sources of novel genetic variation through crosses and as entirely new domesticates targeted for seed products. Many major crops have perennial wild relatives that remain uncharacterized despite their potential.

Nearly all grain and legume crops grown for human consumption are annual plants that complete their life cycle in a single year, or are perennial species cultivated as annuals (Van Tassel et al., 2010). Although thousands of herbaceous perennial species exist within major crop families (e.g., Ciotir et al., 2016; Ciotir et al., 2019), annual plant species were likely selected during the early stages of domestication due to pre-existing agriculturally favorable traits, e.g., high reproductive yield in a single season and accelerated germination and flowering (Van Tassel et al., 2010). Over time, artificial selection has led to exceptional gains in reproductive output in annual crops, particularly in the last century (Mann, 1997); however, cultivation intensity and other agronomic practices have resulted in widespread soil loss (FAO, 2019). Current research focuses in part on the development of crops to support the ecological intensification of agriculture, which aims to achieve both high yields and ecosystem services, such as soil and water retention (Ryan et al., 2018).

The use of herbaceous perennial species as seed crops has been proposed as one method of achieving ecological intensification (Jordan and Warner, 2010; Ryan et al., 2018). Longer-lived species have deep, persistent root systems that mitigate erosion and enhance nutrient uptake, they produce perennating shoots which reduce yearly planting costs, and they have a longer photosynthetically active growth period, allowing for high biomass production each year (Cox et al., 2006; Crews et al., 2018). However, only a few perennial seed crops (principally cereals, oilseeds, and pulses) have entered the domestication process, and we know relatively little about how artificial selection for increased seed production will impact the rest of the perennial plant.

Artificial selection is the human-mediated evolutionary process that leads to changes in plant traits over the course of generations. This process happens as a result of selective cultivation, the act of preferentially planting individuals with desired features. Over time cultivated populations evolve in response to artificial selection, leading to domestication: the evolution of morphological and genetic changes in cultivated populations relative to their wild progenitors (Harlan, 1995; Meyer et al., 2012). Because domestication is an ongoing, evolutionary process, a continuum of cultivated populations exist in species undergoing domestication, ranging from cultivated populations which display little or no differences relative to their wild (uncultivated) progenitors, to highly modified elite breeding lines, such contemporary maize, which differs dramatically from its closest wild relatives (Harris, 1989; Bharucha and Pretty, 2010; Breseghello and Coelho, 2013). To determine the extent to which domestication has occurred, the precise identity of the wild ancestor of a crop and the exact cultivated populations derived from it are required. Because these data are not available for all species examined here, we use the term “cultivated” to refer to any accession that has a history of cultivation, acknowledging that this encompasses a broad spectrum of phenotypic and genetic change under artificial selection.

The “domestication syndrome” describes a common suite of trait changes seen across different species in response to artificial selection for seed and/or fruit production (Hammer, 1984; Harlan, 1992). In annual species, the domestication syndrome typically includes loss of seed dormancy, higher germination, loss of shattering, greater seed size or number, and erect, determinate growth, among many others (Harlan et al., 1973; Olsen and Wendel, 2013; Abbo et al., 2014). In contrast to annuals, the domestication syndrome of woody perennials, such as fruit and nut trees, is characterized by an extended juvenile phase, outcrossing mating system, and often clonal propagation; consequently, woody perennials have typically undergone fewer cycles of sexual selection under domestication and retain a greater proportion of wild genetic diversity relative to annual domesticates (Zohary and Spiegel-Roy, 1975; Miller and Gross, 2011; Gaut et al., 2015). Herbaceous perennial plants cultivated for both edible seeds and sustained perennation were only recently targeted for selection (Suneson et al., 1963; DeHaan et al., 2016; Kane et al., 2016; Kantar et al., 2016; Crews and Cattani, 2018), and it is unclear if they will follow an evolutionary trajectory similar to domesticated annual and woody perennial species, or if they will show a unique domestication syndrome. 

The agricultural context provides an entirely new adaptive landscape that may allow novel combinations of traits in herbaceous perennial species (e.g., high reproductive output and longevity), combinations that are often unfavorable in natural environments (Crews and DeHaan, 2015; Cox et al., 2018). Ongoing work seeks to understand how artificial selection for increased seed yield in herbaceous perennial species might impact vegetative traits and the capacity for perennation more generally. One hypothesis is that perennial seed crops are constrained by a vegetative-reproductive trade-off, where high reproductive allocation and sufficient storage allocation for perennation cannot coexist (Van Tassel et al., 2010). In other words, it may be possible for artificial selection to drive increases in seed yield in wild, herbaceous perennial species, but those increases may cause losses in allocation to vegetative and perennating structures, resulting in a shift from perenniality to annuality (Denison, 2012; Smaje, 2015). Some studies have supported such a trade-off (e.g., González-Paleo et al., 2016; Vico et al., 2016; Cattani, 2017; Pastor-Pastor et al., 2018). An alternative hypothesis is that reproductive yield and vegetative biomass may be selected for in concert, leading to sustained perennation. Concomitant perennation and high seed yield have been observed in some perennial cereals (Sacks et al., 2007; Jaikumar et al., 2012; Culman et al., 2013; Huang et al., 2018).

Herbaceous perennial seed crop studies can benefit from including comparisons with closely related annual domesticates, to clarify if domestication responses are dependent upon lifespan. Vico et al. (2016), in a meta-analysis of 67 annual-perennial pair studies across nine plant families, found greater reproductive allocation in annual crops and greater root allocation in perennial crops, which was the same pattern found in unselected wild groups. Since a majority of the studies in this meta-analysis were from the grass family, such annual-perennial meta-analyses may be augmented by empirical research on specific lineages of plants, to determine if phylogenetically focused trends are similar to the broader patterns observed, as well as to allow a more precise biological interpretation.

Here we focus on the legume family (Fabaceae), which includes 19,500+ species of which more than 30% are predominantly herbaceous perennials (Ciotir et al., 2019). Fabaceae is the second most economically important plant family after the grasses (Poaceae), with 41 domesticated species dating back to the first agricultural systems, and 1,000+ species cultivated for various purposes across the world (Harlan, 1992; Lewis et al., 2005; Hammer and Khoshbakht, 2015). To date, few herbaceous perennial legume species have been thoroughly assessed for agriculturally relevant traits such as seed size, germination rate, time to maturity, root/shoot allocation, and reproductive yield. Characterizing these and similar traits in herbaceous perennial crops and their wild relatives will be critical to assess what genetic variation is available for crop improvement through introgression and *de novo* domestication (Schlautman et al., 2018; Smýkal et al., 2018).

**Figure 1:**
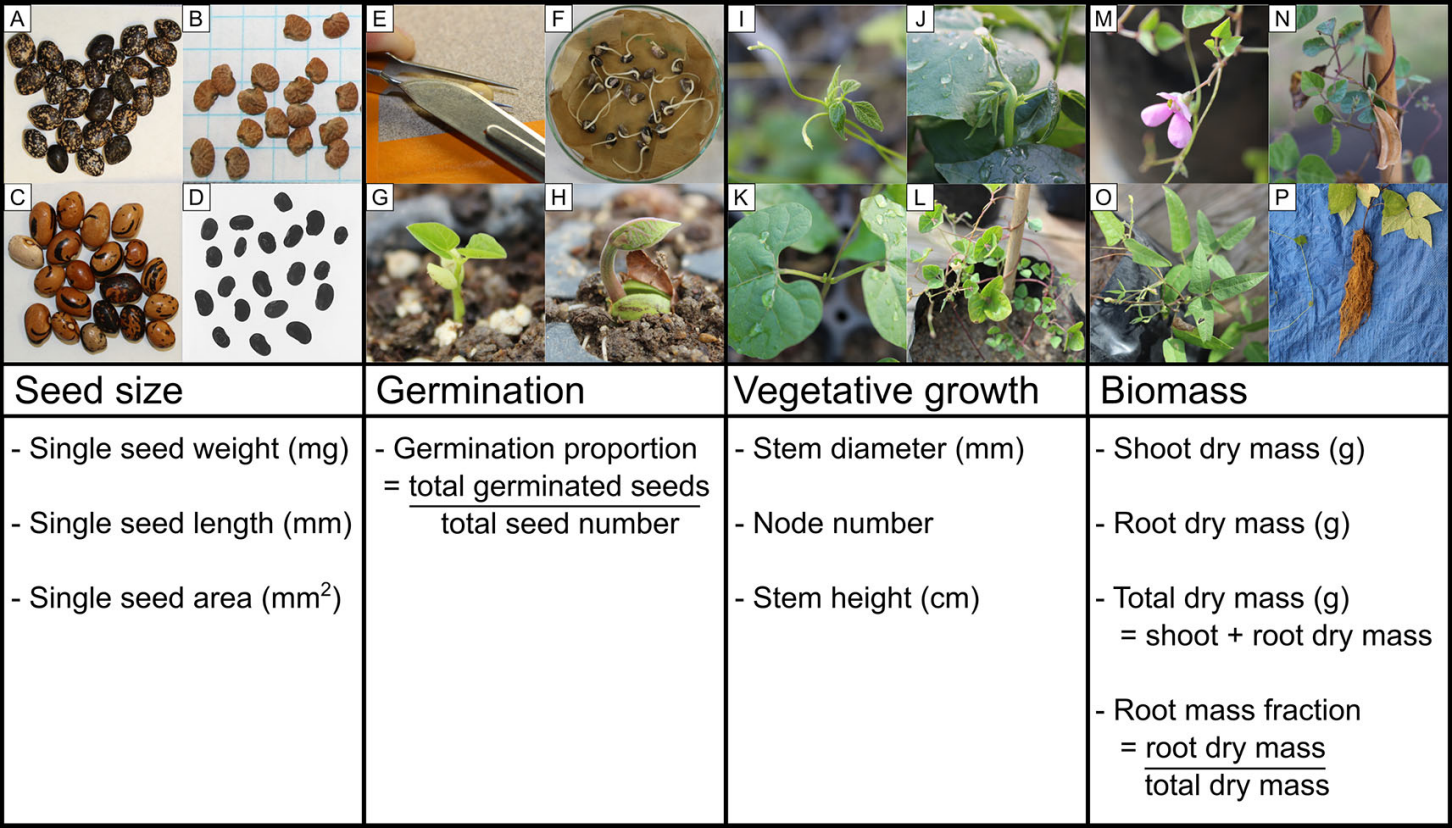
Schematic of the four developmental stages analyzed in this study (seed size, germination, early vegetative growth, and biomass harvest) and examples of phenotypic diversity across the *Phaseolus* species studied: seeds of **(A)** *Phaseolus acutifolius*, **(B)** *P. angustissimus*, and **(C)** *P. coccineus*; **(D)** *P. coccineus* seeds prepared for analysis in ImageJ; **(E)** scarification by nicking the seed coat; **(F)** *P. vulgaris* germinants; **(G)** epigeal germination of *P. filiformis*; **(H)** hypogeal germination of *P. coccineus*; **(I, J)** *Phaseolus* shoot apex at which stem height was measured to; **(K)** *Phaseolus* first node (unifoliate) below which stem diameter was measured; **(L)** fully grown *P. filiformis* shoot with developed nodes (unfolded leaves); **(M, N)** *P. filiformis* flower and ripe fruit; **(O)** *P. acutifolius* shoot biomass; **(P)** *P. coccineus* root biomass. Photo credit: SH.

While there are known consistent differences in growth and resource allocation in some groups of annual and perennial species, gaps remain in our knowledge about how herbaceous perennials respond to artificial selection relative to their annual congeners in many plant lineages. Here we explore life history differences in members of the common bean genus *Phaseolus* by addressing the following questions: 1) How do *wild* annual and perennial *Phaseolus* species from multiple geographic origins allocate resources to seed production and vegetative growth? 2) What is the phenotypic signature of artificial selection in *cultivated* annual and perennial *Phaseolus* species? 3) What covariation exists between seed and adult vegetative growth traits, and is it consistent across lifespans and between cultivated and wild forms in *Phaseolus*? We address these questions by examining seed size, germination, and vegetative growth allocation among three annual and four perennial *Phaseolus* species (**Figure 1**). Through this work, we hope to contribute to ongoing efforts characterizing life history differences in closely related annuals and perennials within Fabaceae and shed light on how these lifespan groups may change with artificial selection.

## Methods

### Plant Material

Three annual *Phaseolus* species (*P. acutifolius, P. filiformis, P. vulgaris*; 58 accessions) and four perennial *Phaseolus* species (*P. angustissimus, P. coccineus, P. dumosus, P. maculatus*; 66 accessions) were included in this study (**Tables 1** and **2**). Species were chosen based on phylogenetic proximity and similar habitat types. The perennials *P. coccineus* and *P. dumosus* and annuals *P. acutifolius* and *P. vulgaris* are in the Vulgaris clade, and the perennial *P. angustissimus* and annual *P. filiformis* are in the Filiformis clade. Perennial *P. maculatus* is the only species in this study from the Polystachios clade (Delgado-Salinas et al., 2006). Geographically, our sampling of *Phaseolus* includes arid-adapted species of the Sonoran Desert (*P. acutifolius*, *P. angustissimus*, *P. filiformis*, and *P. maculatus*) and more tropically distributed species (primarily Mesoamerica: *P. coccineus*, *P. dumosus*, and *P. vulgaris*, with some South American accessions of the latter; **Supplementary Table S4**).

**Table 1:** Summary of sampling for seed size and germination traits for *Phaseolus*, by species and cultivation status.

Lifespan	Species†	Status	Seed size		Germination	
			Accessions	Seeds	Accessions	Seeds
annual	*P. acutifolius*	cultivated	17	25 (20-26)	20	25 (24-26)
		wild	6	25 (25)	10	24 (23-25)
	*P. filiformis*	wild	7	25 (25-26)	7	24 (23-25)
	*P. vulgaris*	cultivated	6	22 (21-24)	6	21 (20-23)
		wild	15	23 (20-30)	15	23 (20-30)
perennial	*P. angustissimus*	wild	2	23 (23)	2	23 (23)
	*P. coccineus*	cultivated	42	20 (19-25)	40	20 (19-25)
		wild	9	25 (20-42)	9	25 (19-41)
	*P. dumosus*	cultivated	8	25 (20-26)	8	24 (20-25)
		wild	2	26 (25-26)	2	23 (20-25)
	*P. maculatus*	wild	3	22 (21-25)	3	21 (21)

† Some species included subspecies designations: P. acutifolius (wild: 3 accessions of var. tenuifolius; cultivated: 2 accessions of var. acutifolius); P. coccineus (wild: 2 accessions of var. coccineus); P. vulgaris (wild: 8 accessions of var. aborigineus); P. maculatus (all accessions of ssp. ritensis). P. vulgaris includes wild accessions from both the Mesoamerican and South American ranges.

‘Accessions’ refers to the total number of accessions used for each species/cultivation status combination. ‘Seeds’ refers to the average seed number among accessions studied for seed size and germination (range of seed number in parentheses). Seed size collectively includes seeds at least measured for length and area, as well as seed weight in all but a few cases.

**Table 2:** Summary of sampling for early vegetative growth and biomass traits for *Phaseolus*, by species and cultivation status.

Lifespan	Species	Status	Stem diameter		Node number		Stem height		Shoot dry mass		Root dry mass		
			Acc.	Plants	Acc.	Plants	Acc.	Plants	Acc.	Plants	Acc.	Plants
annual	*P. acutifolius*	cultivated	3	11 (9-12)	3	11 (9-12)	8	10 (2-25)	9	6 (1-11)	9	1 (1-2)
		wild	5	16 (10-20)	5	15 (6-21)	7	16 (7-24)	6	16 (10-21)	6	4 (2-7)
	*P. filiformis*	wild	7	14 (10-19)	7	14 (10-19)	7	14 (10-19)	7	7 (2-12)	7	2 (1-5)
	*P. vulgaris*	cultivated	5	7 (3-14)	5	7 (3-14)	2	5 (4-6)	4	7 (2-12)	4	2 (1-2)
		wild	12	13 (2-22)	12	13 (2-22)	9	13 (2-22)	12	9 (2-15)	12	2 (1-4)
perennial	*P. angustissimus*	wild	2	7 (6-7)	2	7 (6-7)	2	7 (6-8)	2	2 (2)	1	2
	*P. coccineus*	cultivated	10	9 (2-11)	10	9 (2-11)	13	9 (2-16)	14	7 (1-14)	12	2 (1-4)
		wild	5	9 (2-15)	6	8 (1-15)	4	12 (9-15)	5	7 (1-11)	5	2 (1-3)
	*P. dumosus*	cultivated	1	2	1	2	3	3 (1-5)	2	3 (2-4)	2	1 (1)
	*P. maculatus*	wild	3	8 (2-18)	3	8 (2-17)	2	10 (2-18)	3	6 (3-11)	3	2 (1-3)

“Acc.” refers to the total number of accessions used for each species/cultivation status combination. “Plants” refers to the average plant number among accessions studied for that trait (range of plant number in parentheses).

In total, we obtained seeds from 124 accessions from the United States Department of Agriculture's National Plant Germplasm System (Western Regional PI Station, Pullman, WA, stored at -18°C) in spring 2016, which were stored in a desiccator at 4°C, 33-50% relative humidity. All seeds were derived from plants regrown from the original collection material at the germplasm facility, but there were nevertheless possible genotype by environment and maternal effects that cannot be resolved here. 2,759 seeds were germinated and a subset of these grown from July to September 2016. The seed age for each accession, i.e., the length of time they were in frozen storage, ranged from one year to greater than 46 years (coded as 46).

### Lifespan and Cultivation Status Assignment

We classified species in terms of the predominant lifespan observed in wild populations from their native range. In some cases, this differed from lifespan assignment in the USDA accession description, in which case it was confirmed by extensive literature review; this occurred for *P. coccineus*, *P. dumosus*, and *P. filiformis*. Wild *P. coccineus* is a vigorous, perennial, indeterminate vine with an extensive root system; perenniality is also maintained in many cultivated forms (Delgado-Salinas, 1988; Smartt, 1988; Debouck, 1992; Freytag and Debouck, 2002 *). P. dumosus*, a hybrid of *P. coccineus* and *P. vulgaris*, is also perennial, although it is less frost tolerant than *P. coccineus* (Smartt, 1988; Schmit and Debouck, 1991; Debouck, 1992; Freytag and Debouck, 2002; Mina-Vargas et al., 2016). Lastly, *P. filiformis* is an ephemeral, annual vine primarily found in the Sonoran Desert, which can survive up to seven months in favorable conditions (Buhrow, 1983; Nabhan and Felger, 1985; Freytag and Debouck, 2002). In addition, one accession of *P. maculatus* (PI 494138) was labeled as annual, although the species in general and and other accessions of this species are classified as perennial (Freytag and Debouck, 2002).

Cultivation status was taken directly from the USDA's description, with the descriptors “cultivated,” “cultivar,” and “landrace” all categorized as “cultivated” for the purpose of this data set. We use the umbrella term “cultivated” rather than “domesticated,” since we do not have the data to determine the extent to which phenotypic and genetic change has occurred from the original wild population selected upon (see Introduction). All *Phaseolus* species are native to the Americas and were originally cultivated in either Mesoamerica or South America with some later selection occurring in Eurasia (Bitocchi et al., 2017). Cultivated *Phaseolus* species included here were first domesticated at least 1000 years before present, with most being domesticated much earlier (Kaplan and Lynch, 1999). Species-specific details on geographic origin and domestication are available in **Supplementary Table S4**.

### Traits Measured

#### Seed Size

A total of 51 annual accessions (1,227 seeds) and 66 perennial accessions (1,436 seeds) were analyzed for size traits (**Figure 1**; **Table 1**). Seeds from each accession were weighed in bulk to the nearest mg and mean single seed weight was estimated by dividing the bulk weight by the total number of seeds for that accession. We imaged all accessions on a light table with a fixed camera at a resolution of 640 × 360 or 1349 × 748 pixels (differences accounted for in linear models), with all seeds oriented on their lateral side. From these two-dimensional images, we used ImageJ (Rasband, 1997-2016) to measure mean single seed length and area.

#### Germination

58 annual accessions (1,390 seeds) and 64 perennial accessions (1,369 seeds) were monitored for germination (sampling differences from seed size are due to a few germinated accessions not being analyzed for seed size and vice versa, and some seed loss in the germination procedure). Number of germinated seeds was monitored for each accession and used to calculate germination proportion (**Figure 1**; **Table 1**). Seeds were germinated on RO-water dampened germination paper or with a dampened cotton ball in petri dishes, following 1) sterilization by soaking in 1% bleach for 2 minutes and rinsing, 2) scarification by nicking the seed coat with a scalpel (to break physical dormancy, i.e., a water-impermeable seed coat), and 3) soaking for an average of 23 hours (range: 13-32 hours) by submersion in RO water. The soaking start date ranged from June 26 to July 8, 2016 (one accession soaked on June 19); this was considered time point 0 (i.e., the sowing date) for germination counts, since all necessary resources were available for the seeds to germinate. The germination apparatus was placed onto a 24°C heat mat in 24-hour dark conditions (except for germinant counts and planting; López Herrera et al., 2001). Germination was defined as an extension of the radicle past the seed coat. In rare cases where the seed coat was lost or very hard, it was defined as a distinct vigorous movement of the radicle away from the seed or a distinct pushing of the seed coat away from the seed, respectively. Petri dishes were treated with Banrot 40WP fungicide solution (prepared according to the product label). Fungus-infected, potentially salvageable seeds were soaked in 1% to 2% bleach and rinsed with RO water. Germinated seeds were also scored for seed quality following any potential damage from pre-germination treatments (0-2, with 0 being no damage and 2 being the highest damage), and seeds which were compromised due to procedural damage or had prematurely germinated in storage were removed from the analysis. Germination was monitored prior to planting; accessions with remaining seeds were checked once more 7–10 days after planting for any new germinants. Any variation in germination protocol was noted and addressed in statistical models, as well as the covariates seed quality and seed age (years of frozen storage at the germplasm center since the last seed increase). Subsets of individuals from the original number germinated were chosen for further growth measurement based on the presence of ≥10 vigorous individuals, if the accession was from the native range of the species (preferred), and if the accession was from a duplicate geographic location (removed).

#### Vegetative Growth Measurements

495 annual individuals (40 accessions) and 224 perennial individuals (29 accessions) of *Phaseolus* were transplanted to a greenhouse and measured for at least one vegetative trait (details below; **Figure 1**; **Table 2**). Seedlings were initially planted on July 13–14, 2016 in a mixture of unsterilized local riverine soil (Smoky Hill River, Salina, KS: 38.765948 N, -97.574213 W), sand, and potting soil (PRO-MIX) in small trays until they could be planted in 8” tall x 4” wide bag pots after 2-3 weeks. Initial planting date in small trays was used as the baseline for future measurements (days after planting, DAP), since despite different sowing dates, they were developmentally similar upon planting. Bag pots were filled with a mixture of the same riverine soil and coarse sand, to mimic field soil while also maximizing drainage. All *Phaseolus* were twining and were trained up four-foot bamboo poles. Plants did not receive any rhizobial inoculant treatment. Plants were initially bottom-watered twice daily (10AM, 6PM) for 20 minutes, and a shade cloth was incorporated in the greenhouse. After measurement of early vegetative growth (before biomass), some modifications were made. On August 18, 2016, watering was changed to once for 10 minutes every two days, and the shade cloth was removed on August 23, 2016. 90 of the most vigorous *Phaseolus* plants were moved from the greenhouse to the outdoors on August 25-26, 2016 to expose them to a more light-intense, natural environment. At this time, individual plants in both the greenhouse and outdoors were randomized to reduce spatial bias. All growth analyses were conducted at the research facilities of The Land Institute (Salina, KS).

At 19-23 DAP (25-40 days after sowing), plants were measured for early vegetative growth traits, which included stem diameter below the first node, total developed node number counted from the unifoliate node to the last node with an unfolded leaf, and stem height from ground to shoot apex on the tallest main stem, with twining stems being uncoiled from their poles and straightened as far as possible without damaging the plant. Plants were checked for reproductive status before biomass harvest. At 68-75 DAP (74-93 days after sowing), a random subset of plants was harvested for shoot and root (washed) biomass (**Figure 1**; **Table 2**), which was dried at a minimum of 37°C for at least 24 hours. Biomass was weighed on a precision or analytical scale depending on plant size. Root mass fraction was calculated on an individual plant basis from biomass measurements (root dry mass/total dry mass). Variation in growth conditions (greenhouse or outdoors), plant health (ordinal rating: 0,1,2; unhealthy, moderate health, healthy), and reproductive status (ordinal rating: 0,1,2,3; no reproduction, budding, flowering, fruiting) were recorded for individual plants and accounted for in statistical analyses (see below).

### Statistical Analyses

In order to assess associations of lifespan and cultivation status with trait variation, we used linear models and *post hoc* comparative analyses on a set of mean values for each trait from each accession (**Table 3**). Associations of lifespan, cultivation (nested within lifespan), and species (nested within lifespan and cultivation), in addition to any relevant covariates for the focal trait, were tested using linear models. All potentially confounding factors were included in the original model and were then dropped sequentially if found to be nonsignificant. The base model for all main analyses was: *trait = lifespan + lifespan/cultivation + lifespan/cultivation/species*. Analyses were calculated for accession-level means for all traits and covariates. Due to concerns about lifespan lability of *P. dumosus*, linear models for all traits were checked with this species included and then with the species removed from the dataset. Accessions with any uncertainty associated with their lifespan or cultivation status were also dropped from the model and checked in the same manner; these included PI 494138 (*P. maculatus*; called annual while usually perennial—see Lifespan Assignment) and PI 390770 (*P. vulgaris*, noted as “wild or naturalized” in the USDA description). Pairwise comparisons of lifespan and cultivation effects for each trait were evaluated with *post-hoc* Tukey HSD tests (**Supplementary Table S1**).

**Table 3:** Results of linear models for all traits in the total *Phaseolus* dataset.

	Trait	Lifespan	Cultivation	Species			
(a)	Seed weight	*F*_1_ = 99.07***	*F*_2_ = 38.11***	*F*_7_ = 1.28			
	Seed length†	*F*_1_ = 162.81***	*F*_2_ = 69.20***	*F*_7_ = 3.59**			
	Seed area†	*F*_1_ = 124.61***	*F*_2_ = 41.76***	*F*_7_ = 1.47			
	**Trait**	**Lifespan**	**Cultivation**	**Species**	**Seed age**	**Soak time**	**Seed quality**
(b)	Germination proportion	*F*_1_ = 3.10	*F*_2_ = 3.15*	*F*_7_ = 1.84	*F*_1_ = 2.43	*F*_1_ = 1.72	*F*_1_ = 13.69***
	**Trait**	**Lifespan**	**Cultivation**	**Species**	**Health**		
(c)	Stem diameter	*F*_1_ = 4.03	*F*_2_ = 36.82***	*F*_6_ = 3.44**	*F*_1_ = 0.24		
	Node number	*F*_1_ = 20.00***	*F*_2_ = 7.13**	*F*_6_ = 4.99***	*F*_1_ = 17.53***		
	Stem height	*F*_1_ = 1.94	*F*_2_ = 2.22	*F*_6_ = 1.51	*F*_1_ = 10.99**		
	**Trait**	**Lifespan**	**Cultivation**	**Species**	**Health**	**Reproductive status**	**Outdoor proportion**
(d)	Shoot dry mass	*F*_1_ = 21.25***	*F*_2_ = 18.71***	*F*_6_ = 2.43*	*F*_1_ = 2.08	*F*_1_ = 1.80	*F*_1_ = 5.00*
	Root dry mass	*F*_1_ = 11.80**	*F*_2_ = 10.53***	*F*_6_ = 1.37	*F*_1_ = 8.35**	*F*_1_ = 3.57	*F*_1_ = 0.69
	Total dry mass	*F*_1_ = 14.44***	*F*_2_ = 14.47***	*F*_6_ = 2.02	*F*_1_ = 7.33**	*F*_1_ = 3.49	*F*_1_ = 0.43
	Root mass fraction	*F*_1_ = 3.33	*F*_2_ = 4.92*	*F*_6__=_ 4.89***	*F*_1_ = 13.89***	*F*_1_ = 0.09	*F*_1_ = 4.15*

*P < 0.05; **P < 0.01; ***P < 0.001.

†Differences in image resolution (640x360 or 1349×748 pixels) were accounted for in this model: F values were nonsignificant (0.0566 for seed length and 0.8301 for seed area).

Letters denote separate models with different covariates, while the main effects are the same for all traits: (a) seed size traits, (b) germination proportion, (c) early vegetative growth traits, and (d) biomass traits. See the Methods for explanations of each covariate.

We ran separate linear models and Tukey HSD tests for *Phaseolus* wild accessions to detect any phenotypic signatures of geographic origin, divided broadly into desert-adapted and tropical-adapted species (**Supplementary Tables S2** and **S3**). The base model for the geographic analyses was: *trait = geography + lifespan + geography×lifespan + geography/lifespan/species*, including any relevant covariates. Cultivated accessions were not included in this model due to potentially confounding effects of artificial selection. See **Supplementary Table S4** for the assignment of geographic origin categories to species.

To assess trait covariation within the whole dataset, Pearson product-moment correlations were performed on all pairwise combinations of the 11 measured traits using mean accession-level data, to determine the magnitude and direction of their relationships. Pearson correlations were also run on the following subsets of the data to qualitatively assess any trait covariation differences: annual, perennial, cultivated, and wild (**Supplementary Figure S1**). Each subset of the data was only restrictive in regard to one criterion, e.g., the annual subset contained data for both cultivated and wild annual accessions. Statistical analyses and figure generation were performed in R v. 3.6.1 (R Core Team, 2019).

All accession-level and individual plant data can be found in **Supplemental Table S5**. All individual seed size data may be found in **Supplemental Table S6**.

## Results

**Figure 2:**
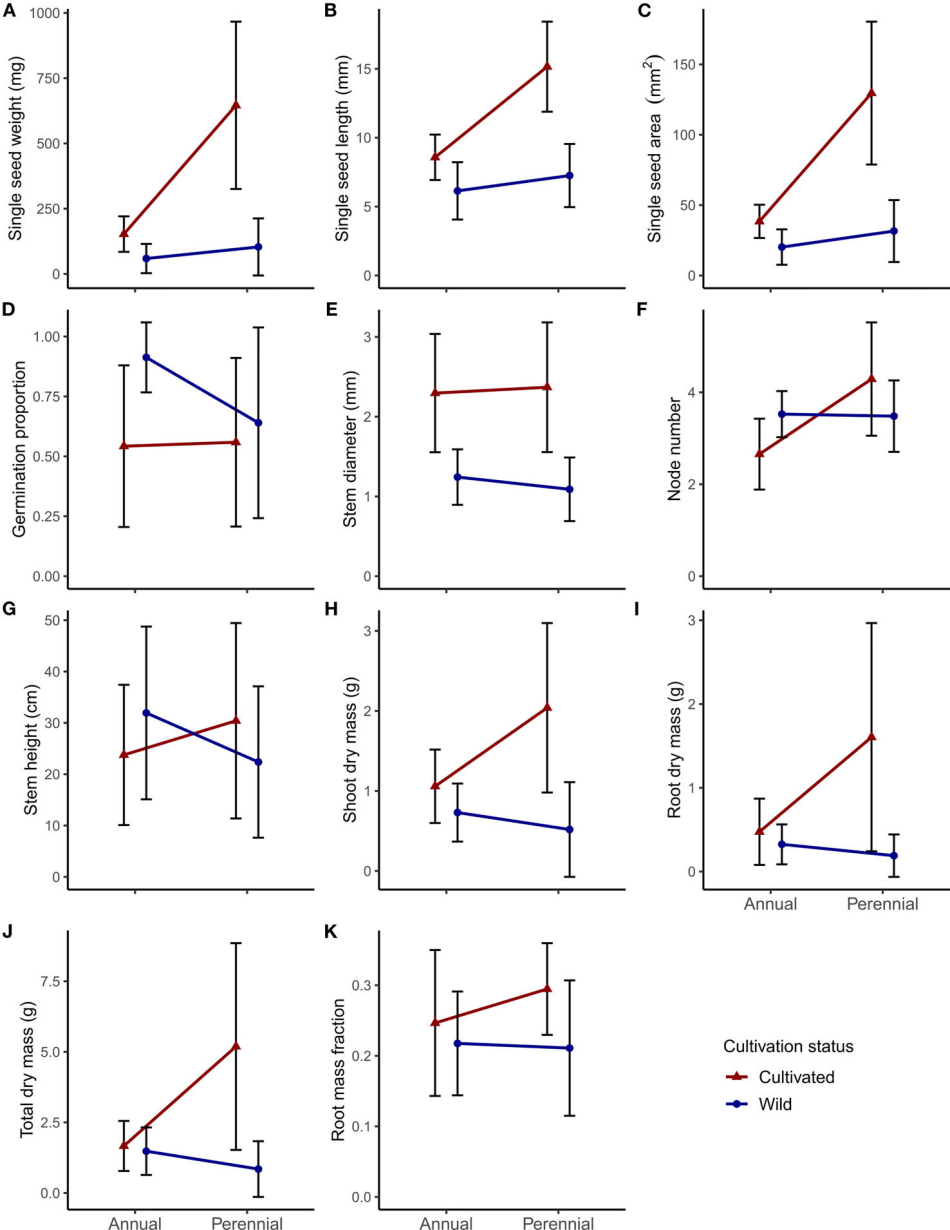
Panel of phenotypic differences between lifespan (annual or perennial) and cultivation status (cultivated or wild) for the 11 focal traits in *Phaseolus*: **(A–C)** seed size traits; **(D)** germination proportion; **(E–G)** early vegetative growth traits; **(H–K)** shoot and root biomass traits. Central points represent means of all accessions for that category; error bars represent one standard deviation.

**Figure 3:**
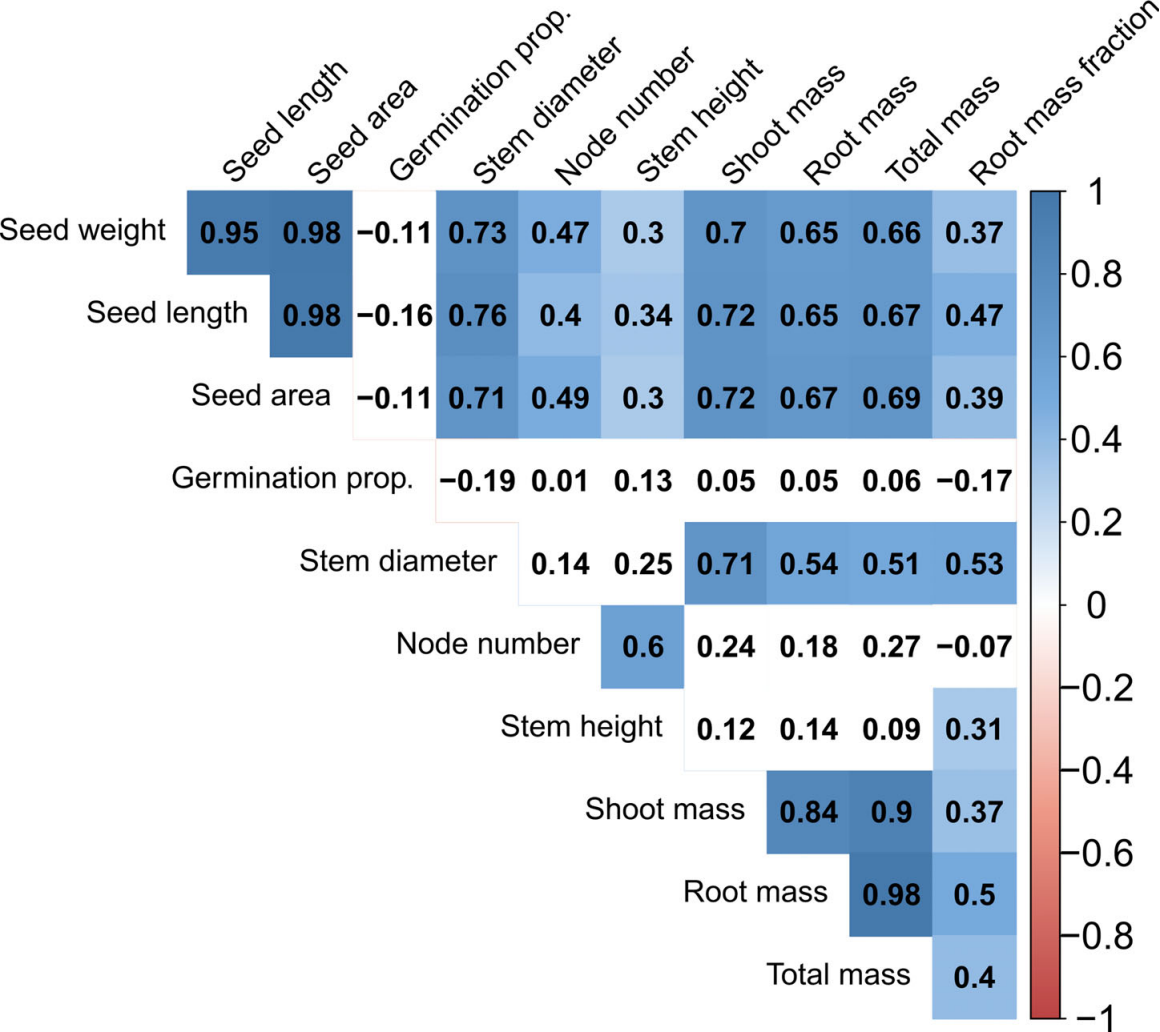
Correlation diagram of all traits for all accessions in the *Phaseolus* dataset. Numbers in boxes represent the Pearson correlation coefficient. Blue and red colors indicate significant positive and negative correlations (at *P* < 0.05), respectively; absence of color indicates lack of significance.

We investigated annual and perennial *Phaseolus* species for potential differences and covariation in seed size, germination, and vegetative growth. We found that wild accessions of annual species showed nonsignificantly greater germination and vegetative trait values than wild perennial accessions, but did not show greater seed size traits (**Figure 2**). Cultivated accessions of both annual and perennial species had greater trait values compared to wild accessions for almost all traits measured, with greater mean increases observed in perennial species (**Figure 2**). Lastly, seed and vegetative traits were significantly positively correlated, with some variation in this trend within subsets of the data (**Figure 3**; **Supplementary Figure S1**).

### Trait Differences in Wild Annual vs. Perennial *Phaseolus* Accessions

Although nonsignificant, wild perennial *Phaseolus* mean seed weight was nearly twice that of wild annuals (59 mg annual vs. 104 mg perennial; **Figure 2A**; see **Supplementary Table S1** for all mean values, standard deviation, and Tukey test significance). Wild annual germination proportion was nonsignificantly higher than that of wild perennials (0.91 annual vs. 0.64 perennial; **Figure 2D**; **Supplementary Table S1**). Wild annual *Phaseolus* had similar to nonsignificantly larger vegetative trait values compared to wild perennials, with the largest relative differences seen in root dry mass (0.32 g annual vs. 0.19 g perennial) and total dry mass (1.48 g annual vs. 0.85 g perennial; **Figures 2I, J**; **Supplementary Table S1**). Mean root mass fraction was nearly equivalent for wild annuals (0.22) and wild perennials (0.21; **Figure 2K**; **Supplementary Table S1**).

In our main linear models, lifespan explained a significant amount of the variation seen in all seed size traits, node number, and most biomass traits (except root mass fraction; **Table 3**). Seed age and soak time were not significant predictors of germination; seed quality had significance at *P* < 0.001 (**Table 3**). Plant health was significant (at least at *P* < 0.01) in the linear models for all vegetative growth and biomass traits except stem diameter and shoot dry mass (**Table 3**). Reproductive status was not significant for any biomass trait; outdoor proportion was significant at *P* < 0.05 for shoot dry mass and root mass fraction (**Table 3**). The removal of *P. dumosus* and the accessions PI 494138 (*P. maculatus*) and PI 390770 (*P. vulgaris*; see Methods) changed some linear model results; here we note traits for which any variable's significance was lost or gained in the main model, which occurred for some vegetative traits. The exclusion of *P. dumosus* resulted in lifespan becoming significant for stem diameter (*P* < 0.05), in species becoming nonsignificant for shoot dry mass, and in outdoor proportion becoming nonsignificant for root mass fraction. Similarly, the exclusion of PI 494138 resulted in lifespan becoming significant for stem diameter (*P* < 0.05) but also for root mass fraction (*P* < 0.05). The exclusion of PI 390770 did not change significance in any of our models.

While our data do not allow precise interpretation of geographic effects, linear models including geographic origin as a main effect along with lifespan found that geographic origin explained a significant portion of the variation seen in all seed size traits and most vegetative traits for wild *Phaseolus* accessions (**Supplementary Table S2**). In general, tropically distributed *Phaseolus* accessions had larger seed and vegetative growth traits compared to desert species (**Supplementary Table S3**). Mean germination proportion was relatively high (> 0.85) for all *Phaseolus* groups except tropical perennials (0.48, although tropical perennials also had the greatest standard deviation; **Supplementary Table S3**).

### Trait Differences in Cultivated vs. Wild *Phaseolus* Accessions

Cultivated annual and perennial *Phaseolus* accessions showed generally larger seed and vegetative size characteristics compared to wild relatives (**Figures 2**). Cultivation differences in seed size were only significant for seed length in annual *Phaseolus*, although cultivated annual seed weight (153 mg) was nearly three times larger than wild annuals (59 mg) (**Figure 2A**; **Supplementary Table S1**). Cultivated perennial *Phaseolus* had significantly greater seed size in all traits, with seed weight over six times larger in cultivated perennials (646 mg) than wild perennials (104 mg) (**Figure 2A**; **Supplementary Table S1**). Germination proportion was significantly lower in cultivated annual *Phaseolus* (0.54) relative to wild annuals (0.91), while cultivated perennial germination proportion was only slightly lower (0.56) than wild perennials (0.64) (**Figure 2D**; **Supplementary Table S1**).

Cultivated perennial *Phaseolus* accessions tended to have significantly larger vegetative features compared to their wild relatives, whereas cultivated annual *Phaseolus* accessions usually displayed nonsignificantly larger vegetative features compared to their wild relatives (**Figures 2E–K**; **Supplementary Table S1**). Stem diameter was however significantly greater in both cultivated annual and perennial *Phaseolus* (**Figure 2E**; **Supplementary Table S1**). Cultivated annual *Phaseolus* showed nonsignificantly lower values in node number and stem height compared to wild annuals, while cultivated perennial *Phaseolus* showed nonsignificantly larger values in both traits compared to wild perennials (**Figures 2F, G**; **Supplementary Table S1**). Both cultivated annual and perennial *Phaseolus* had greater dry biomass trait values than their wild relatives, but for cultivated annual *Phaseolus*, the only significantly larger biomass value was shoot dry mass (1.06 g cultivated vs. 0.73 g wild; **Figure 2H**; **Supplementary Table S1**). In contrast, all biomass trait values were significantly greater in cultivated relative to wild perennial *Phaseolus* (except root mass fraction), with a greater than six-fold larger root dry mass value (1.60 g cultivated vs. 0.19 g wild; **Figure 2I**; **Supplementary Table S1**). In our linear models, cultivation explained a significant amount of variation for all traits except stem height (**Table 3**).

### Phenotypic Covariation Across All Traits

Trait covariation was predominantly positive within the total dataset (**Figure 3**) and each subset of the data (**Supplementary Figures S1A-D**). Considering the entire dataset, all seed dimensions (weight, length, area) were nearly perfectly correlated (R^2^ = 0.95-0.98; **Figure 3**). Seed traits were also significantly positively correlated with all vegetative growth traits, having the highest correlation with stem diameter and most biomass traits (R^2^ = 0.65-0.76; **Figure 3**). Stem diameter had positive albeit non-significant correlations with node number and stem height, and significant positive correlations with all biomass traits. Node number and stem height had nonsignificant positive correlations with most biomass traits (**Figure 3**). Biomass traits including shoot dry mass, root dry mass, total dry mass, and root mass fraction were significantly positively correlated with one another, and there was a tight relationship between shoot and root dry mass (R^2^ = 0.84; **Figure 3**). Germination proportion was the only trait in the total dataset to have no significant correlations and to have more than two negative correlations with other traits (**Figure 3**).

Subsets of our data also displayed similarly positive trait correlations with some exceptions. Due to sample size, each subset was restricted in regard to only one criterion (lifespan or cultivation status), e.g., the perennial subset contained both cultivated and wild perennial accessions. Notably, the annual subset showed negative correlations between node number and most traits (significant for seed size traits, stem diameter, and shoot dry mass), as well as a significant negative correlation of germination proportion with seed weight and stem diameter (**Supplementary Figure S1A**). In contrast, the perennial, cultivated, and wild subsets respectively tended to show positive correlations for the same trait pairings which were negatively correlated in annuals, although germination proportion and node number showed at least one negative correlation in all subsets (**Supplementary Figure S1B-D**). Significant positive seed size to vegetative trait correlations tended to be stronger and more common in the perennial and cultivated subsets than the annual and wild subsets (**Supplementary Figures S1**).

## Discussion

This study examined lifespan and cultivation effects among annual and herbaceous perennial legume species in the economically important genus *Phaseolus*, with the aim of identifying common trends and potential covariation among traits. Consistent trends included greater seed and plant size in cultivated relative to wild accessions for both annual and perennial species, and positive correlations among most seed and vegetative traits for annual and perennial species in the wild and under cultivation.

### Wild Annual and Perennial *Phaseolus* Accessions Show Only Slight Differences in Trait Values

Wild perennial *Phaseolus* accessions in this study exhibited somewhat greater seed size trait values relative to wild annual accessions, which is consistent with the general life history expectation that later successional species produce larger seeds that are better able to compete for resources (Thompson and Hodkinson, 1998). This trend was consistent for *Phaseolus* species native to the desert and the tropics (**Supplementary Table S3**). Tropical *Phaseolus* species had higher mean seed size than desert *Phaseolus*, which is consistent with the broad pattern of increasing seed size at lower latitudes (Moles et al., 2007; **Supplementary Table S3**). The large range of phenotypic variation observed in annual and perennial *Phaseolus* (**Figures 2A–K**) is also consistent with the large distributions of several species, such as *P. vulgaris* and *P. coccineus,* which inhabit environments ranging from very arid to very humid (Dohle et al., 2019).

While few studies have compared germination in wild *Phaseolus* species, Bayuelo-Jiménez et al. (2002) reported a similarly high germination proportion (0.85+) for scarified wild annual and perennial *Phaseolus* accessions, including the desert species *P. angustissimus* and *P. filiformis*. Although wild annuals showed higher mean germination than wild perennials considering the whole dataset, desert perennial accessions all reached 100% germination, suggesting that the broader trend is more applicable to the tropical species studied here (**Supplementary Table S3**). Our lowest wild mean germination was observed in the tropical perennials (*P. coccineus* and *P. dumosus*; **Supplementary Table S3**), which may reflect the trend that seed dormancy is less common in tropical herbaceous species in regions of higher rainfall (Baskin and Baskin, 2014).

While germination showed consistent differences between wild annuals and perennials, we cannot make ecologically robust conclusions here, since we effectively removed the legumes' primary form of physical dormancy through scarification, and each accession was stored frozen for different lengths of time at USDA facilities, which can have diverse effects on species' germination biology (Walters et al., 2005). Our results may be more reflective of the viability of seeds after long-term storage. In this light, our findings are consistent with life history predictions that annual species will maintain a complex, long-lived seed bank in which dormancy breaking and germination is staggered over time to ensure offspring survival in variable environments (Venable and Lawlor, 1980; Gremer et al., 2016). Perennials may be less selectively constrained by this pressure due to the parent plant's persistent survival in more stable environments (Cohen, 1966; Thompson et al., 1998).

The lack of significant differences in vegetative traits between wild annual and perennial *Phaseolus* suggests that the traits studied here do not diverge at this growth stage among different *Phaseolus* life history strategies in nature. Wild perennials' slightly lower mean vegetative growth could be due to a slower growth rate and the early stage of growth at which the traits were measured (three to eleven weeks after planting), since some perennials have been shown to be able to achieve a higher total biomass than related annuals when the entire growing season is considered (Dohleman and Long, 2009). Previous studies have also found that vegetative growth, shoot biomass, and root biomass are similar between closely related annuals and perennials, up to 40 days of growth, before their resource allocation patterns diverge (De Souza and Vieira Da Silva, 1987; Garnier, 1992). The significance of plant health in vegetative linear models for stem height and node number may reflect a greater sensitivity of response in stem length traits to environmental stressors compared to stem diameter; the health effect on root dry mass and derived traits (total dry mass and root mass fraction) may be due to a lower sample size for these traits compared to shoot dry mass alone (**Tables 2** and **3**).

### Cultivated *Phaseolus* Accessions Show Greater Seed and Plant Size Relative to Wild Accessions

*Phaseolus* species showed larger seed and vegetative trait values in cultivated relative to wild accessions, consistent with domestication syndrome expectations; however, there were some notable differences in this trend between the annual and perennial groups. Consistent with previous studies in *Phaseolus* (Smartt, 1988; Koinange et al., 1996; Aragao et al., 2011), all cultivated annuals and perennials exhibited greater seed size relative to wild accessions. Perennial *Phaseolus* showed a particularly large difference in cultivated vs. wild seed size, as well as substantial variation in this trait, suggesting a large amount of genetic diversity. Much of the perennial seed size variation stemmed from *P. coccineus,* which is primarily outcrossing and more genetically diverse than other *Phaseolus* crops (Bitocchi et al., 2017; Guerra-García et al., 2017). Our seed size observations in USDA accessions are consistent with an analysis of wild and domesticated *Phaseolus* accessions from the International Center for Tropical Agriculture (CIAT), with the exception that they observed larger ranges of seed weight in cultivated annual species (*P. acutifolius*, *P. vulgaris*; Chacón-Sánchez, 2018). They similarly found that the perennial *P. coccineus* had the largest range in seed size in both cultivated and wild accessions, that *P. coccineus* showed the largest mean seed weight increase with cultivation, and that the perennial *P. dumosus* also showed some of the largest cultivated seed weights (Chacón-Sánchez, 2018).

Cultivation effects on *Phaseolus* germination may reflect different annual-perennial seed dormancy strategies. Domestication in annual common bean (*P. vulgaris*) and other seed crops has been known to reduce seed coat thickness and seed dormancy (Koinange et al., 1996; Fuller and Allaby, 2009), which could expose cultivated accessions to premature water imbibition and seed mortality in storage (López Herrera et al., 2001). Comparably low germination proportion in cultivated and wild perennial *Phaseolus* could be due to a less selective pressure for seed dormancy (and therefore lower seed longevity) in wild perennials relative to wild annuals, resulting in less change in germination proportion with domestication. Since our accessions were of various ages and geographic origin, more precise studies on fresh seedstock are necessary to confirm germination differences with cultivation for both annual and perennial species.

Vegetative size was generally larger in cultivated relative to wild annuals and perennials, although there were some notable exceptions. Mean node number and stem height were lower in cultivated annual *Phaseolus* and higher in cultivated perennial *Phaseolus* relative to wild accessions. Similarly, node number and height have been found to be lower in cultivated relative to wild annual *P. vulgaris* (Koinange et al., 1996; Berny Mier y Teran et al., 2018). This suggests that on average cultivated annual *Phaseolus* exhibit lower degrees of stem growth, which may be the result of direct selection for determinate growth or an indirect consequence of increased harvest index, i.e., biomass allocated to reproductive structures at the expense of vegetative growth. This is in spite of all of the cultivated accessions in this study retaining their twining habit. In contrast, cultivated perennial *Phaseolus* exhibited consistently higher values for vegetative growth relative to wild perennials, possibly indicating a persistence of indeterminate growth in cultivation (Smartt, 1988), allowing for simultaneous selection on increased vegetative growth and reproduction (but see Delgado-Salinas, 1988). Both cultivated annual and cultivated perennial accessions displayed higher shoot and root dry mass relative to wild accessions, in agreement with the findings of Berny Mier y Teran et al. (2018) in *P. vulgaris*; this coincided with higher values in stem diameter for cultivated accessions of both lifespans, in agreement with Smartt (1988). Under cultivation, higher shoot and root biomass relative to wild accessions have also been observed in 30 diverse annual crop species, attributed to greater seed size and leaf area allowing enhanced downstream effects on growth (Milla et al., 2014; Milla and Matesanz, 2017). Our study suggests that some cultivated perennial species in *Phaseolus* exhibit similar size increases.

Root mass fraction, a resource-conservative trait, exhibited higher values in cultivated relative to wild accessions for both lifespans; this contradicts the expectation that plants become more resource-acquisitive with domestication (McKey et al., 2012; Vilela and González-Paleo, 2015; Pastor-Pastor et al., 2018). Higher root mass fraction was also observed in the annuals *Phaseolus vulgaris* (Berny Mier y Teran et al., 2018) and *Pisum sativum* (Weeden, 2007) relative to wild forms. This difference could be a byproduct of a more general higher allocation to vegetative growth, allowing greater amounts of photosynthate to be allocated to roots (Weeden, 2007). In summary, while many vegetative traits exhibit higher values in cultivated relative to wild accessions for both annual and perennial *Phaseolus* species, some traits may exhibit divergent lifespan patterns. It remains to be determined if this is due to lifespan *per se* or each species' unique history of artificial selection.

### Trait Covariation Is Significantly Positive in Most Cases

Positive correlations among traits observed in our dataset suggest that some suites of traits are synergistic, including seed size, some early growth traits, and biomass allocation (**Figure 3**). Positive seed-vegetative size correlations have also been found in other herbaceous plant systems (Geber, 1990; Kleyer et al., 2019), as well as across vascular plants more generally (Díaz et al., 2015). The nearly perfect correlation among seed size parameters (weight, area, and length) suggests that both two-dimensional seed features and mass are jointly selected upon and can be used interchangeably for these species. Significant positive correlations between seed size and vegetative biomass are consistent with the fundamental constraint of plant size on seed size, i.e., small plants cannot produce very large seeds (Venable and Rees, 2009). Furthermore, positive correlations between stem diameter and seed size support the hypothesis that seed size is biomechanically limited by the size of the subtending branch (Aarssen, 2005; Venable and Rees, 2009). Seed size alone may be less reflective of whole-plant reproductive allocation (Vico et al., 2016) and more so of a tolerance-fecundity tradeoff, where larger seeds are produced in fewer numbers but have greater competitive ability in stressful conditions (Muller-Landau, 2010). Nevertheless, the perennial grain crop *Thinopyrum intermedium* showed a positive relationship of total biomass and single seed mass with total reproductive yield across three years of growth, suggesting that these traits are positively correlated in some perennial species (Cattani and Asselin, 2018; also see Kleyer et al., 2019). Lastly, significant positive associations between all dry biomass traits do not support aboveground-belowground tradeoffs; rather, the data suggest that larger plants require greater resource acquisition with proportionally larger roots. This is consistent with a previous study reporting positive correlations among shoot dry mass, root dry mass, and root mass fraction in *Phaseolus vulgaris* (Berny Mier y Teran et al., 2018).

There were a few notable exceptions to the general trend of positive correlations. There were unexpectedly no significant correlations between seed size and germination proportion considering the entire dataset, with only slight negative correlations detected. There is a theoretical expectation that germination dormancy and seed bank persistence are negatively related to seed size, since dormancy and seed size entail different strategies of surviving in different environmental conditions, i.e., small, dormant seeds dominate seasonal environments and large, non-dormant seeds are typical of aseasonal environments (Baskin and Baskin, 2014; Rubio de Casas et al., 2017). The annual and wild subsets showed greater negative seed size-germination correlations (significant for germination vs. seed weight for annuals), contrasting with positive correlations in the perennial and cultivated subsets, suggesting some association of lifespan and cultivation with this trait relationship (**Supplementary Figure S1**). The removal of the physical dormancy barrier through scarification must also be considered, although we may still expect differences in seed longevity in storage proportional to seed size.

There was also a general lack of significant covariation between early vegetative stem growth traits (stem height and node number) and most biomass traits (**Figure 3**). This is in contrast to a previous study reporting that stem height is a highly interconnected hub trait in herbaceous perennial species (Kleyer et al., 2019), although we find many of the same positive associations between vegetative and seed size traits. The significant negative correlations detected in annual species between seed size traits and node number may reflect tradeoffs between seed size and stem length allocation due to the biomechanical restraints of bearing larger seeds on a longer stem (Kleyer et al., 2019) or may be the result of reduced stem growth during artificial selection (see above discussion), although this correlation was positive in all other data subsets (**Supplementary Figure S1**).

Overall, positive correlations among traits measured here suggests that future breeding efforts targeting greater seed size in perennials may be possible without concomitant reductions in vegetative allocation. Consistently high correlations may also allow for measurement of simpler traits (e.g., stem diameter, shoot dry mass) as proxies for traits that are more difficult to assess (e.g., root dry mass, total yield).

## Conclusion

For this set of *Phaseolus* species, we found that cultivation is associated with an increase in seed size and overall vegetative size in both annuals and perennials, and that seed size and most vegetative traits were positively correlated in both cultivated and wild annuals and perennials. Traits are shaped and predicted by more specific factors than lifespan alone, including habitat differences, evolutionary history, and which specific organs were targeted during artificial selection. Resolving these factors will require a focused sample of close sister species from the same native range, or intraspecific ecotypes of differing lifespan. Also, all perennials in our study were analyzed in their first year of growth, while their overall life history strategy is contingent upon their survival across multiple years. Thus, this study may also be augmented by multiyear assessments of reproductive and vegetative trait variation in perennial individuals. Such studies will advance our basic knowledge of life history evolution and inform plant breeding as we determine viable methods of ecological intensification. Conclusively, we highlight here common features in cultivated annual and perennial species compared to their wild relatives, and we observed few tradeoffs among seed and vegetative traits in the first year of growth. This offers insight into how perennial legume crops may respond to artificial selection relative to their annual relatives, and it suggests that many traits of interest may be selected for in concert.

## Data Availability Statement

All datasets generated for this study are included in the article/**Supplementary Material**.

## Author Contributions

AM and SH designed the project. SH implemented the project and wrote the manuscript, with significant input from AM and MR. MR assisted in statistical analysis and figure generation. CC, TC, and DT provided extensive technical assistance, helpful feedback on the basis and design of the study, and revisions to the manuscript. All authors read and approved the final version of the manuscript.

## Funding

This research was funded by the Perennial Agriculture Project (Malone Family Land Preservation Foundation and The Land Institute). SH is supported by a graduate assistantship from Saint Louis University. MR is supported by the Donald Danforth Plant Science Center and the Perennial Agriculture Project.

## Conflict of Interest

The authors declare that the research was conducted in the absence of any commercial or financial relationships that could be construed as a potential conflict of interest.
