# Supplementary material for: Comparative Analysis of Early Life Stage Traits in Annual and Perennial Phaseolus Crops and Their Wild Relatives
Source: Front Plant Sci. 2020 Mar 10;11:34. doi: 10.3389/fpls.2020.00034 (PMC7076113; doi:10.3389/fpls.2020.00034)
Supplement: Supplementary file 1 [file Image_1.pdf]

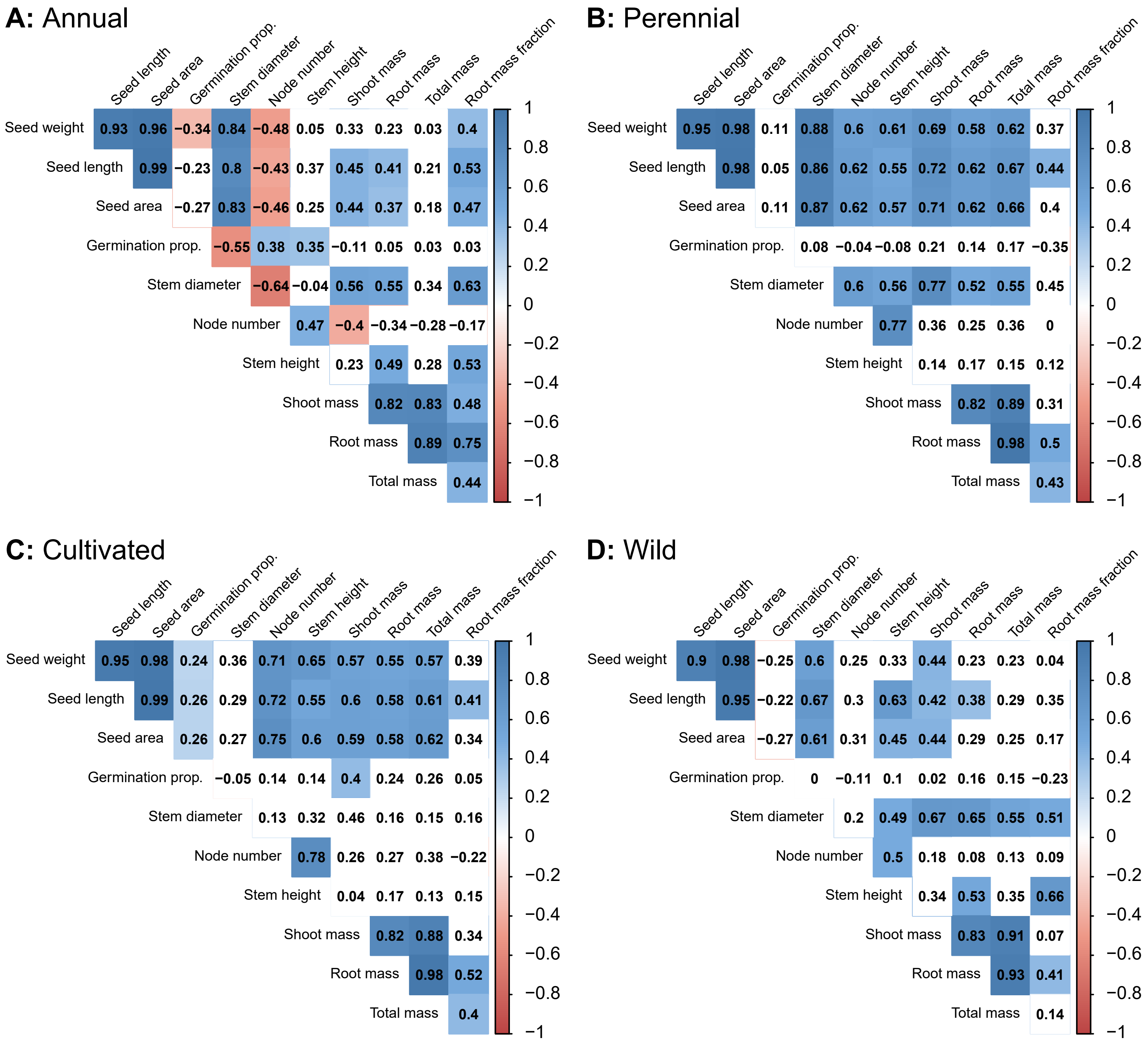

Figure S1. Correlation diagrams of all traits for subsets of the *Phaseolus* dataset: (A) annual accessions, (B) perennial accessions, (C) cultivated accessions, and (D) wild accessions. Each category includes all other data which match the criterion (e.g., 'annual' includes both cultivated and wild annual accessions). Numbers in boxes represent the Pearson correlation coefficient. Blue and red colors indicate significant positive and negative correlations (at $P < 0.05$), respectively; absence of color indicates lack of significance.
