# Supplementary material for: Comparative Analysis of Early Life Stage Traits in Annual and Perennial Phaseolus Crops and Their Wild Relatives
Source: Front Plant Sci. 2020 Mar 10;11:34. doi: 10.3389/fpls.2020.00034 (PMC7076113; doi:10.3389/fpls.2020.00034)
Supplement: Supplementary file 2 [file Table_1.docx]

**Table S1.** Mean seed and vegetative trait values for *Phaseolus* species, with one standard deviation, grouped by lifespan and cultivation status. Different letters indicate a significant difference (at least *P* < 0.05) within a trait between lifespan × cultivation groups according to a *post-hoc* Tukey HSD test run on the linear model, with covariates included.

|  | Group | | | |
| --- | --- | --- | --- | --- |
| Trait | cultivated annual | wild annual | cultivated perennial | wild perennial |
| Single seed weight (mg) | 152.83 ± 67.96 ^A^ | 59.24 ± 55.94 ^A^ | 645.94 ± 320.56 ^B^ | 103.56 ± 109.35 ^A^ |
| Single seed length (mm) † | 8.58 ± 1.65 ^A^ | 6.14 ± 2.08 ^B^ | 15.15 ± 3.27 ^C^ | 7.25 ± 2.29 ^AB^ |
| Single seed area (mm^2^) † | 38.45 ± 11.82 ^A^ | 20.19 ± 12.58 ^A^ | 129.59 ± 50.76 ^B^ | 31.58 ± 22.00 ^A^ |
| Germination proportion | 0.54 ± 0.34 ^A^ | 0.91 ± 0.15 ^B^ | 0.56 ± 0.35 ^A^ | 0.64 ± 0.40 ^AB^ |
| Stem diameter (mm) | 2.30 ± 0.74 ^A^ | 1.24 ± 0.35 ^B^ | 2.37 ± 0.81 ^A^ | 1.09 ± 0.40 ^B^ |
| Node number | 2.66 ± 0.77 ^A^ | 3.53 ± 0.50 ^AB^ | 4.29 ± 1.23 ^B^ | 3.48 ± 0.78 ^AB^ |
| Stem height (cm) | 23.76 ± 13.66 ^A^ | 31.94 ± 16.83 ^A^ | 30.41 ± 19.04 ^A^ | 22.38 ± 14.74 ^A^ |
| Shoot dry mass (g) | 1.06 ± 0.46 ^AB^ | 0.73 ± 0.36 ^C^ | 2.04 ± 1.06 ^A^ | 0.52 ± 0.59 ^BC^ |
| Root dry mass (g) | 0.47 ± 0.40 ^A^ | 0.32 ± 0.24 ^A^ | 1.60 ± 1.36 ^B^ | 0.19 ± 0.25 ^A^ |
| Total dry mass (g) | 1.67 ± 0.89 ^A^ | 1.48 ± 0.84 ^A^ | 5.19 ± 3.66 ^B^ | 0.85 ± 0.99 ^A^ |
| Root mass fraction | 0.25 ± 0.10 ^AB^ | 0.22 ± 0.07 ^A^ | 0.29 ± 0.07 ^B^ | 0.21 ± 0.10 ^AB^ |

† Image resolution is also accounted for in these calculations.
