# Supplementary material for: Comparative Analysis of Early Life Stage Traits in Annual and Perennial Phaseolus Crops and Their Wild Relatives
Source: Front Plant Sci. 2020 Mar 10;11:34. doi: 10.3389/fpls.2020.00034 (PMC7076113; doi:10.3389/fpls.2020.00034)
Supplement: Supplementary file 3 [file Table_2.docx]

**Table S2.** Results of linear models used to assess broad geographic effects on lifespan-related seed and vegetative trait variation in wild *Phaseolus*. Letters denote separate models with different covariates, while the main effects are the same for all traits: (a) seed size traits, (b) germination proportion, (c) early vegetative growth traits, and (d) biomass traits. See the Methods for explanations of each covariate.

|  | Trait | Geography | Lifespan | Geography *×* Lifespan | Species |  |  |  |
| --- | --- | --- | --- | --- | --- | --- | --- | --- |
| (a) | Seed weight | *F*_1_ = 13.49*** | *F*_1_ = 2.31 | *F*_1_ = 0.53 | *F*_3_ = 1.24 |  |  |  |
|  | Seed length† | *F*_1_ = 36.80*** | *F*_1_ = 2.01 | *F*_1_ = 1.81 | *F*_3_ = 1.42 |  |  |  |
|  | Seed area† | *F*_1_ = 27.97*** | *F*_1_ = 4.91* | *F*_1_ = 2.04 | *F*_3_ = 0.54 |  |  |  |
|  | Trait | Geography | Lifespan | Geography *×* Lifespan | Species | Age | Soak time | Seed quality |
| (b) | Germination probability | *F*_1_ = 3.89 | *F*_1_ = 6.92* | *F*_1_ = 10.10** | *F*_3_ = 0.23 | *F*_1_ = 0.71 | *F*_1_ = 0.01 | *F*_1_ = 2.42 |
|  | Trait | Geography | Lifespan | Geography *×* Lifespan | Species | Health |  |  |
| (c) | Stem diameter | *F*_1_ = 12.81** | *F*_1_ = 1.85 | *F*_1_ = 0.18 | *F*_2_ = 0.65 | *F*_1_ = 0.14 |  |  |
|  | Node number | *F*_1_ = 0.00 | *F*_1_ = 0.24 | *F*_1_ = 8.50** | *F*_2_ = 1.48 | *F*_1_ = 7.45* |  |  |
|  | Stem height | *F*_1_ = 8.57** | *F*_1_ = 1.94 | *F*_1_ = 0.85 | *F*_2_ = 3.58* | *F*_1_ = 1.84 |  |  |
|  | Trait | Geography | Lifespan | Geography *×* Lifespan | Species | Health | Reproductive state | Outdoor proportion |
| (d) | Shoot dry mass | *F*_1_ = 5.71* | *F*_1_ = 1.68 | *F*_1_ = 1.77 | *F*_2_ = 0.82 | *F*_1_ = 1.98 | *F*_1_ = 0.33 | *F*_1_ = 0.00 |
|  | Root dry mass | *F*_1_ = 10.00** | *F*_1_ = 3.26 | *F*_1_ = 0.08 | *F*_2_ = 0.38 | *F*_1_ = 2.33 | *F*_1_ = 0.04 | *F*_1_ = 3.01 |
|  | Total dry mass | *F*_1_ = 4.47* | *F*_1_ = 3.71 | *F*_1_ = 0.51 | *F*_2_ = 0.20 | *F*_1_ = 1.98 | *F*_1_ = 0.02 | *F*_1_ = 0.81 |
|  | Root mass fraction | *F*_1_ = 16.13*** | *F*_1_ = 0.07 | *F*_1_ = 4.26 | *F*_2_ = 0.79 | *F*_1_ = 3.35 | *F*_1_ = 0.50 | *F*_1_ = 3.84 |

**P* < 0.05; ***P* < 0.01; ****P* < 0.001

† Differences in image resolution (640x360 or 1349×748 pixels) were accounted for in this model: F values were nonsignificant (1.5636 for seed length and 4.0726 for seed area).
