# Supplementary material for: Comparative Analysis of Early Life Stage Traits in Annual and Perennial Phaseolus Crops and Their Wild Relatives
Source: Front Plant Sci. 2020 Mar 10;11:34. doi: 10.3389/fpls.2020.00034 (PMC7076113; doi:10.3389/fpls.2020.00034)
Supplement: Supplementary file 4 [file Table_3.docx]

**Table S3.** Mean seed and vegetative trait values for wild *Phaseolus* species, with one standard deviation, grouped by lifespan and broad geographic distribution. Different letters indicate a significant difference (at least *P* < 0.05) within a trait between lifespan × geography groups according to a *post-hoc* Tukey HSD test run on the linear model, with covariates included.

|  | Group | | | |
| --- | --- | --- | --- | --- |
| Trait | desert annual | desert perennial | tropical annual | tropical perennial |
| Single seed weight (mg) | 20.89 ± 14.83 ^A^ | 32.94 ± 18.36 ^A^ | 89.92 ± 58.05 ^AB^ | 135.65 ± 119.06 ^B^ |
| Single seed length (mm) † | 4.47 ± 1.10 ^A^ | 5.19 ± 1.33 ^A^ | 7.59 ± 1.58 ^B^ | 8.19 ± 2.02 ^B^ |
| Single seed area (mm^2^) † | 11.06 ± 5.74 ^A^ | 14.56 ± 7.32 ^AB^ | 28.11 ± 11.51 ^B^ | 39.32 ± 22.22 ^B^ |
| Germination proportion †† | 0.86 ± 0.18 ^A^ | 1.00 ± 0.00 ^AB^ | 0.97 ± 0.05 ^A^ | 0.48 ± 0.38 ^B^ |
| Stem diameter (mm) | 1.05 ± 0.15 ^AB^ | 0.84 ± 0.23 ^A^ | 1.44 ± 0.38 ^C^ | 1.34 ± 0.38 ^BC^ |
| Node number | 3.56 ± 0.44 ^A^ | 3.00 ± 0.86 ^A^ | 3.49 ± 0.58 ^A^ | 3.88 ± 0.44 ^A^ |
| Stem height (cm) | 23.50 ± 13.01 ^A^ | 9.37 ± 3.29 ^A^ | 45.06 ± 13.63 ^B^ | 35.38 ± 6.73 ^AB^ |
| Shoot dry mass (g) | 0.66 ± 0.33 ^A^ | 0.17 ± 0.14 ^A^ | 0.81 ± 0.39 ^A^ | 0.87 ± 0.68 ^A^ |
| Root dry mass (g) | 0.21 ± 0.14 ^A^ | 0.06 ± 0.06 ^A^ | 0.45 ± 0.26 ^B^ | 0.30 ± 0.31 ^AB^ |
| Total dry mass (g) | 1.26 ± 0.59 ^A^ | 0.26 ± 0.17 ^A^ | 1.68 ± 1.00 ^A^ | 1.32 ± 1.14 ^A^ |
| Root mass fraction | 0.16 ± 0.05 ^A^ | 0.20 ± 0.09 ^AB^ | 0.27 ± 0.05 ^B^ | 0.22 ± 0.11 ^AB^ |

† Image resolution is also accounted for in these calculations.

†† The age and soak time covariates had to be included separately in the Tukey test due to the limits of this subset of the data; both models produced the same Tukey results.
