# Supplementary material for: Comparative Analysis of Early Life Stage Traits in Annual and Perennial Phaseolus Crops and Their Wild Relatives
Source: Front Plant Sci. 2020 Mar 10;11:34. doi: 10.3389/fpls.2020.00034 (PMC7076113; doi:10.3389/fpls.2020.00034)
Supplement: Supplementary file 5 [file Table_4.docx]

**Table S4.** Summary of each *Phaseolus* species’ native distribution in the wild form, cultivated uses, and timeline of its earliest estimated cultivation. This represents the overall description of each species and does not necessarily reflect all accessions used in this study. Vegetable refers either to vegetative parts or green pods consumed by humans. A blank signifies no known cultivated uses (not counting ethnobotanical uses).

| **Lifespan** | **Species** | **Native distribution**† | **Cultivated uses** | **Original cultivation**†† | **Citations** |
| --- | --- | --- | --- | --- | --- |
| Annual | *P. acutifolius* | SW United States to Mexico (D) | pulse | 500 BCE | Buhrow 1983; Smartt 1988; Kaplan & Lynch 1999; Bitocchi et al. 2017 |
|  | *P. filiformis* | SW United States to Mexico (D) |  |  | Buhrow 1983 |
|  | *P. vulgaris* | W Mesoamerica and W South America (T) | pulse; vegetable | 3030 BCE | Smartt 1988; Kaplan & Lynch 1999; Bitocchi et al. 2017 |
| Perennial | *P. angustissimus* | SW United States to Mexico (D) |  |  | Buhrow 1983 |
|  | *P. coccineus* | Mexico to Panama (T) | pulse; vegetable | 880 CE | Smartt 1976; Delgado-Salinas 1988; Smartt 1988; Kaplan & Lynch 1999 |
|  | *P. dumosus* | Guatemala (T) | pulse; vegetable | ††† | Smartt 1988; Schmit & Debouck 1991 |
|  | *P. maculatus* | SW United States to Mexico (D) |  |  | Buhrow 1983 |

† Capital letters denote the broad geographic distribution assigned to each species which was used in linear models: D refers to desert and T to tropical.

†† Evidence for the earliest cultivation in *Phaseolus* species is generally highly variable, ranging from 9000 BCE to less than 500 years ago; here we use more recent radiocarbon dates taken directly from *Phaseolus* material, but we recognize that this is dependent upon material that was carbonized or did not decay significantly (Kaplan & Lynch 1999).

††† To our knowledge, the timing and precise nature of *Phaseolus dumosus* domestication has not yet been determined.

**References for Table S4:**

Bitocchi, E., Rau, D., Bellucci, E., Rodriguez, M., Murgia, M. L., Gioia, T., et al. (2017). Beans (*Phaseolus* ssp.) as a model for understanding crop evolution. *Frontiers in Plant Science*, 8, 722. doi: 10.3389/fpls.2017.00722

Buhrow, R. (1983). The wild beans of southwestern North America. *Desert Plants*, 5(2), 67–88

Delgado-Salinas, A. (1988). Variation, taxonomy, domestication, and germplasm potentialities in *Phaseolus coccineus*. In P. Gepts (Ed.), *Genetic Resources of Phaseolus Beans* (pp. 441–463). Norwell, MA: Kluwer Academic Publishers. doi: 10.1007/978-94-009-2786-5_18

Kaplan, L., & Lynch, T. F. (1999). *Phaseolus* (Fabaceae) in archaeology: AMS radiocarbon dates and their significance for pre-Colombian agriculture. *Economic Botany*, 53(3), 261–272. doi: 10.1007/BF02866636

Schmit, V., & Debouck, D. G. (1991). Observations on the origin of *Phaseolus polyanthus* Greenman. *Economic Botany*, 45, 345–364. doi: 10.1007/BF02887077

Smartt, J. (1976). Comparative evolution of the pulses. *Euphytica* 25, 139-143. doi: 10.1007/BF00041538

Smartt, J. (1988). Morphological, physiological, and biochemical changes in *Phaseolus* beans under domestication. In P. Gepts (Ed.), *Genetic Resources of Phaseolus Beans* (pp. 143–161). Norwell, MA: Kluwer Academic Publishers. doi: 10.1007/978-94-009-2786-5_8
